# Associations between Schistosomiasis and the Use of Human Waste as an Agricultural Fertilizer in China

**DOI:** 10.1371/journal.pntd.0003444

**Published:** 2015-01-15

**Authors:** Elizabeth J. Carlton, Yang Liu, Bo Zhong, Alan Hubbard, Robert C. Spear

**Affiliations:** 1 Department of Environmental and Occupational Health, Colorado School of Public Health, University of Colorado Anschutz Medical Campus, Aurora, Colorado, United States of America; 2 Institute of Parasitic Diseases, Sichuan Center for Disease Control and Prevention, Chengdu, China; 3 Division of Biostatistics, School of Public Health, University of California, Berkeley, California, United States of America; 4 Center for Occupational and Environmental Health, School of Public Health, University of California, Berkeley, California, United States of America

## Abstract

**Background:**

Human waste is used as an agricultural fertilizer in China and elsewhere. Because the eggs of many helminth species can survive in environmental media, reuse of untreated or partially treated human waste, commonly called night soil, may promote transmission of human helminthiases.

**Methodology/Principal Findings:**

We conducted an open cohort study in 36 villages to evaluate the association between night soil use and schistosomiasis in a region of China where schistosomiasis has reemerged and persisted despite control activities. We tested 2,005 residents for *Schistosoma japonicum* infection in 2007 and 1,365 residents in 2010 and interviewed heads of household about agricultural practices each study year. We used an intervention attributable ratio framework to estimate the association between night soil use and *S. japonicum* infection. Night soil use was reported by half of households (56% in 2007 and 46% in 2010). Village night soil use was strongly associated with human *S. japonicum* infection in 2007. We estimate cessation of night soil use would lead to a 49% reduction in infection prevalence in 2007 (95% CI: 12%, 71%). However, no association between night soil and schistosomiasis was observed in 2010. These inconsistent findings may be due to unmeasured confounding or temporal shifts in the importance of different sources of *S. japonicum* eggs on the margins of disease elimination.

**Conclusions/Significance:**

The use of untreated or partially treated human waste as an agricultural fertilizer may be a barrier to permanent reductions in human helminthiases. This practice warrants further attention by the public health community.

## Author Summary

Many people use human waste as an agricultural fertilizer, often called “night soil.” If the waste is not properly treated, the use of night soil may promote the spread of infectious diseases. We suspected that night soil use may facilitate the spread of the water-borne disease, schistosomiasis, as some schistosomiasis eggs can survive in the environment for weeks. We conducted a study in 36 villages in rural China in order to see if the amount of night soil used in a village was associated with schistosomiasis. The study was conducted in an area where schistosomiasis reemerged and persisted despite an aggressive disease control program. We found half of households reported using night soil—it was used on all major crops and by people across the socio-economic spectrum. We also found that night soil use was strongly associated with schistosomiasis infection in 2007, but not in 2010. Our findings show the use of human waste as an agricultural fertilizer is common in our study region and may increase schistosomiasis infections. The extent to which night soil is used and risks of this practice should be evaluated as part of disease control programs targeting schistosomiasis and other human helminthiases.

## Introduction

For centuries, people have collected human waste and used the material, called *night soil*, to fertilize agricultural crops [1, 2]. The use of human fecal waste as an agricultural fertilizer has the potential to improve crop yields without the expense, environmental risk or transportation infrastructure required of synthetic fertilizers. However, if the waste is not properly treated, the practice may promote fecal-borne diseases [3].

High temperatures, high pH, desiccation and the introduction of additives such as leaf litter and ash can reduce pathogen loads in human waste products, allowing use on agricultural commodities. But fresh human waste or waste that has been stored under suboptimal conditions, may contain human pathogens, presenting risks to individuals that handle night soil and to those who may come into contact with food, soil or water in areas where night soil is applied [3]. Helminth eggs are particularly hardy and may require longer storage and/or higher temperatures and pH for destruction relative to other pathogens found in human stool [4].

The use of night soil may be particularly hazardous in the context of schistosomiasis, a water-borne infection that causes anemia, fibrosis of the liver and kidneys and impairs growth and cognitive development [5, 6]. The eggs of *Schistosoma japonicum*, the parasite that causes intestinal schistosomiasis in Asia, and *S. mansoni*, which causes intestinal schistosomiasis in the Americas, Africa and the Middle East, are excreted in stool. Upon contact with water, eggs hatch into miracidia and then must mature within a snail host before the parasite can infect humans. *Schistosome* eggs can survive for several days (*S. mansoni*) or weeks (*S. japonicum*) in excreted stool [4]. Schistosome host snails thrive in irrigation ditches and rice paddies [7–9]. Thus, the application of human waste to agricultural crops can transport schistosome eggs from stool pits directly to snail habitat, facilitating the schistosome life cycle.

The use of night soil may be a barrier to permanent schistosomiasis control in China. Chinese public health officials are attempting elimination of schistosomiasis using a multi-pronged control effort that includes anti-helminthic treatment, reduction of snail populations and improvements to water and sanitation infrastructure [10]. While schistosomiasis disease burden has declined in China and transmission has been eliminated in five provinces, pockets of transmission persist and reemergence has been detected in previously controlled areas [11–13]. The challenge of sustained interruption of schistosomiasis is increasingly relevant outside of China as endemic countries adopt antihelminthic treatment programs and look towards permanent reductions in schistosomiasis infections and morbidity [14].

The reemergence and persistence of schistosomiasis may depend, in part, on the *internal potential* of an area, the set of conditions that make an area hospitable (or inhospitable) to the schistosome lifecycle such as the availability of snail habitat, human water contact patterns and waste disposal practices [15, 16]. The output of *S. japonicum* eggs into the environment and efforts to reduce egg output through improved sanitation have been previously shown to impact long-term disease patterns in endemic areas [15]. Improving sanitation access is a priority for the schistosomiasis control program in China, and the importance of safe sanitation is recognized by global programs to reduce human helminthiases [10, 14]. However, night soil use has not been a focus of schistosomiasis control activities.

Here, we evaluate the association between night soil use and human schistosomiasis in a region where schistosomiasis reemerged and persisted in the presence of ongoing disease control programs. Because information about the use of human waste as an agricultural fertilizer is limited in the public health literature, we first document night soil practices in 36 villages in rural China. We then test the hypothesis that greater night soil use in a village is associated with increased human *S. japonicum* infection prevalence.

## Methods

### Study population

This research was conducted in two rural counties in Sichuan, China where schistosomiasis reemerged following the reduction of human and bovine infection prevalence below 1%, the Chinese Ministry of Health threshold for transmission control [12]. In 2007, we selected 53 villages in 3 counties where schistosomiasis had reemerged for a longitudinal study of social and environmental determinants of schistosomiasis reemergence [17]. One county was excluded from follow-up because it was severely impacted by the 7.9 magnitude earthquake that struck Sichuan May 12, 2008. The analysis presented here includes the 36 villages in the two counties followed through 2010. The names and exact locations of the study villages have been withheld to protect the privacy of study participants and promote candid reporting.

In rural Sichuan, populations fluctuate due to rural-to-urban migration, as well as marriages, births and deaths. We therefore employed an open cohort design, conducting a census in 2007 and 2010 in order to identify all residents living in the study villages, age 6 and older. All individuals identified in each census were recruited for *S. japonicum* infection testing and household surveys. In 2007, we identified 2,891 eligible residents. In 2010, we identified 2,287 residents, including 1,875 identified in the previous census and 412 new residents. Of the 1,016 people identified in 2007 that were no longer residents in 2010, 760 had left their village for work, 203 had left to attend school, 33 had died and 20 had left for marriage.

The research protocol was approved by the Sichuan Institutional Review Board and the University of California, Berkeley, Committee for the Protection of Human Subjects. All participants provided written, informed consent before participating in this study. All children provided assent and their parents or guardians provided written, informed permission for them to participate in this study. Everyone testing positive for *S. japonicum* was notified and provided treatment with 40 mg/kg praziquantel by the anti-schistosomiasis control station.

### Data collection

**Household interviews**. In the summer of 2007 and 2010, the head of each household was invited to complete a structured interview about agricultural practices, sanitation access and socio-economic status (SES). Survey instruments were pilot tested in the study region and administered by trained public health workers fluent in the local dialect. We interviewed 1,156 households in 2007 and 951 households in 2010.

Participants reported all crops planted in the past 12 months, the quantity of night soil applied to each crop and whether chemical fertilizers were used. The amount of night soil used by each household was calculated as the total quantity of night soil applied to all crops in the past 12 months. An additional metric of night soil use was also developed for supplemental analyses in light of evidence that improved toilet designs reduce the number of viable helminth eggs in effluent [18, 19]. Households with a working anaerobic biogas digester or triple compartment septic tank were classified as having improved sanitation, and night soil applied by such households was classified as coming from improved sanitation sources. Night soil applied by all other households was classified as coming from unimproved sources.

SES was assessed using an asset-based approach. Household assets can provide a more stable estimate of long-term wealth than monetary income in agrarian regions, where income is episodic and includes agricultural products [20, 21]. The index included ownership of eight durable goods (car, motorcycle, tractor, computer, television, air conditioner, washing machine and refrigerator), reported by the head of household. Principal components analysis was used to create an aggregated SES score, deriving weights from the first principal component which explained 28% of the total variance between measures.

Multiple imputation by chained equations was used to impute missing household survey data. Multiple imputation avoids biases due to exclusion of incomplete cases and reduces the variance introduced by the uncertainties of imputation through the generation of multiple datasets [22, 23]. The method assumes that data are missing at random—that missingness can be explained by the other, observed variables used in the imputation. We imputed the quantity of night soil used, the number of bovines owned by the household, SES score, whether the household had an improved toilet and the area of land cultivated by the household. Because the volume of night soil used and the number of bovines per household were highly right-skewed, predictive mean matching was used for the imputation of these variables. We imputed the missing data using the aforementioned variables, including observations from prior/future time points and county of residence, generating 20 imputed datasets. Imputed values were used for the 19% of households missing each of these survey measures, including the 482 households not interviewed and 13 households with incomplete survey data.

***S. japonicum* infection testing**. Everyone identified in each census was invited to submit three stool samples on three consecutive days in November/December of 2007 and 2010 for *S. japonicum* infection testing. Each sample was examined using the miracidium hatching test [24]. Briefly, 30 g of stool were suspended in aqueous solution, strained with copper mesh to remove large particles and strained with nylon mesh to concentrate schistosome eggs. The sediment was re-suspended in water and left undisturbed in a room with ambient temperatures between 28 and 30°C. Samples were examined two, five and eight hours after sample preparation for the presence of miracidia. One stool sample per person was also examined using the Kato-Katz thick smear procedure [25]. Three slides were prepared from each sample, using 41.7 mg stool per slide. Slides were allowed 24 hours to clear and were examined for *S. japonicum* eggs by trained technicians using a dissecting microscope. Stool samples were delivered to county laboratories daily for analysis. A person was classified as infected if any test was positive for *S. japonicum*.

### Estimation of village-level night soil use

Because night soil use by one household may impact the *S. japonicum* infection risk of other village residents, we wanted to include both village- and household-level night soil use in our statistical models. However, using a village-level measure that is an average of all household-level measures results in a given individual’s household night soil use appearing in the model twice—once as part of the average, and again as an individual-level variable. This endogeniety leads to theoretical challenges. Using an outcomes based causal framework, we typically define the effect of an exposure, X, on an outcome as the difference in mean outcomes when the population is uniformly at X = a vs. X = b (where a and b are any combination of exposure levels, only one of which is observable) for some target population with a specific distribution of confounders [26]. But the overlap of household and village-level variables makes it difficult to evaluate changes in one without changes in the other. To avoid this problem, we defined village-level night soil use as the average amount of night soil applied by all households in the village excluding the index household. This allows for theoretical considerations of the effect of changes in village-level night soil use holding an individual’s household-level night soil use constant and vice versa.

### Statistical analysis

We employed a two-step approach to evaluate the relationship between night soil use and human infection. First, we examined the association between village-level night soil use and human *S. japonicum* infection using a multi-level, fixed-effect logistic regression model, modeling village-level night soil use as a categorical variable to allow for non-linear relationships between the explanatory variable and the outcome. Tests for trend were conducted by treating the categorical variable as ordinal. Models were run separately for each study year. Potential confounding variables were selected a priori based on prior evidence and the plausibility of a relationship with both the outcome of interest and night soil use. Models adjusted for participant age, county, household night soil use, bovine ownership, village bovine density (the mean number of bovines per household), area of land cultivated by the household in the past year, agricultural intensity in the village (mean area cultivated in the past year per household), household SES score and village SES (mean household SES score). Village SES, bovine density and agricultural intensity were estimated separately for each household, excluding the index household, as described above. Occupation was not included as a potential confounder, as greater than 95% of adults in the region reported their occupation as farmer in 2007 [17]. Given the large set of potential confounders and the uncertainties in variable selection, we defined a reduced set of variables that we strongly suspected to be confounders (age, sex, county and household-level night soil use) and ran all models twice, once using the reduced set and once using the full set of confounders. We accounted for correlation within villages using generalized estimating equations and exchangeable working correlation, calculating robust variance estimates [27].

Second, we evaluated the potential impact of interventions to reduce the use of night soil on schistosomiasis. To do this, we estimated a parameter akin to attributable risk using a population intervention model approach [28, 29] that we call the intervention attributable ratio. The intervention attributable ratio is defined as E(Y_A_)/E(Y), where E(Y_A_) is the expected prevalence of infection in the study population if night soil use were eliminated, and E(Y) is the observed prevalence of infections at the observed levels of night soil use. For the purpose of the model, we assume that all infections were acquired recently (a reasonable assumption given the frequency of mass and targeted chemotherapy in this population), that our measure of night soil use is relevant to the infection risk period, and that the observed statistical associations between night soil use and schistosomiasis prevalence represent a causal relationship. We explore the strengths and limitations of these assumptions in the discussion. G-computation was used [28–30]. We fit a fixed-effect logistic regression model assuming an independent correlation structure to allow for a population-level prevalence estimate. Based on the results of the first analysis, we included the limited set of potential confounders, modeled each year separately and modeled village night soil use as a continuous variable. This model was used to calculate infection probabilities for each individual surveyed when household and village night soil use were reduced to zero. Inference was estimated by bootstrapping: the population was sampled with replacement by village to obtain a 36 village population, the model was re-fit, E(Y) was estimated as the observed infection prevalence in the resampled population and E(Y_A_) was estimated as the predicted infection prevalence when household and village night soil use were set to zero, as above, repeating this procedure 1,000 times. The 2.5^th^ and 97.5^th^ percentile values used to estimate the 95% confidence interval. All analyses were conducted using Stata version 12 (College Station, TX).

## Results

### Night soil use

Human waste was used as an agricultural fertilizer in 56% and 46% of households surveyed in 2007 and 2010, respectively. Night soil was applied to all major summer and winter crops and by households across SES categories (Table 1). In 2007, 99% of households that planted one or more crops used chemical fertilizers. Night soil was usually gathered from sources within the household: in 2007, 8% of households with improved sanitation and 7% of households without improved sanitation reported using night soil from other households. Night soil was collected from all toilet types. Of those that used night soil in 2010, 91% of households with an anaerobic biogas digester, 94% with a triple compartment septic tank and 96% with an unimproved toilet reported removing waste from their toilet system to use as an agricultural fertilizer.

**Table 1:** The use of human waste as an agricultural fertilizer in 36 villages in Sichuan, China.

	**2007 (n = 1,156)**		**2010 (n = 951)**	
	**No. households**	**Pct. using night soil**	**No. households**	**Pct. using night soil**
Crops planted^a^				
Rapeseed	1007	47	786	25
Rice	875	37	598	22
Wheat	701	30	425	9
Corn	673	54	731	23
Peanuts	221	33	592	8
Vegetables	194	65	503	63
Planting by season^b^				
Summer	1102	51	851	48
Winter	1094	47	821	26
Socio-economic status^c^				
Low	596	61	208	49
Medium	403	52	199	46
High	157	44	544	44
Improved sanitation^d^				
No	965	56	689	44
Yes	190	55	251	49

^a^ Crops planted by at least 10% of households in a given season are listed.

^b^ Rapeseed and wheat are grown in the winter season (typically October to April); rice, corn, vegetables and peanuts are grown in the summer season (typically May to September).

^c^ Socio-economic status was calculated based on ownership of eight durable goods, aggregated using principal components analysis. Categories were defined by tertile.

^d^ Households were classified as having improved sanitation if they had a working anaerobic biogas digester or triple compartment septic tank.

The mean quantity of night soil applied per household declined from 67 buckets per household in 2007 to 32 buckets per household in 2010 (Table 2). Access to improved sanitation increased over the study period, but most night soil applied came from unimproved sources: 79% and 72% of all night soil was applied by households without improved sanitation in 2007 and 2010, respectively.

**Table 2:** Description of study participants in 36 villages in Sichuan, China.

	**2007**	**2010**
**Individuals tested for *S. japonicum* infection**	**n = 2,005**	**n = 1,365**
Positive for *S. japonicum* infection (%)	8.4	7.4
Mean infection intensity (EPG)	2.1	1.3
Male (%)	49.0	46.7
Mean age	42.3	46.4
Live in County 2 (%)	43.1	44.2
**Households surveyed**	**n = 1,156**	**n = 951**
Use night soil	55.5	45.5
Night soil applied in the past year (buckets/household)	67.4	31.9
Have an improved toilet (%)^a^	16.5	26.7
Have a working anaerobic biogas digester (%)	11.1	20.4
Have a trip compartment septic tank (%)	5.8	6.2
Have an unimproved toilet	81.8	72.4
No toilet in the household	1.7	0.9
Use chemical fertilizers (%)^b^	95.0	
Own bovines (%)	45.4	27.7
Mean number of bovines per household	0.58	0.39
Cultivated land in the past year (%)	95.8	90.8
Mean area cultivated in the past year (hectares)	0.3	0.3
Own a car (%)	2.0	7.3
Own a tractor (%)	2.0	1.6
Own a motorcycle (%)	35.0	57.1
Own a computer (%)	0.5	11.3
Own a television (%)	94.1	95.8
Own a washing machine (%)	47.7	75.3
Own an air conditioner (%)	1.5	6.0
Own a refrigerator (%)	12.8	57.7

EPG—Eggs per gram of stool

^a^ Includes households with a working anaerobic biogas digester or a triple compartment septic tank.

^b^ Asked only in 2007.

### Schistosomiasis prevalence and infection intensity

We tested 2,005 people in 36 villages for *S. japonicum* infection in 2007 and 1,365 people in the same villages in 2010 (69% and 60% of the eligible populations identified in the census in 2007 and 2010, respectively). Participation in the infection surveys was higher among older populations and, particularly in 2010, among residents in County 2 (S1 Table). *S. japonicum* infection prevalence was 8.4% in 2007 and 7.4% in 2010. Among infected individuals, infection intensity was low. The mean eggs per gram of stool among infected was 24 in 2007 and 18 in 2010. *S. japonicum* infections were detected in 28 villages in 2007 and 20 villages in 2010.

### The association between night soil use and schistosomiasis

**Categorical analysis**. Village night soil use was associated with increased prevalence of human *S. japonicum* infection in 2007 but not in 2010. Table 3 shows the relationship between night soil use and *S. japonicum* infection when village night soil use was categorized into quartiles. In 2007, individuals in areas where village night soil use was in the highest quartile were 10.8 times more likely to be infected with *S. japonicum* compared to the lowest quartile, adjusting for the full set of potential confounders (95% CI 3.25, 35.87). Infection prevalence increased with village night soil use in 2007 (test for trend, adjusting for the full set of potential confounders, *p* = 0.009). Village night soil use was not associated with *S. japonicum* infection in 2010. Household-level night soil use was not associated with *S. japonicum* infection regardless of year and the set of confounders included in the model. Estimates and inference were similar using the limited vs. full set of confounders. Estimates and inference were also similar when we limited the analyses to the 1,577 individuals in 2007 and 747 individuals in 2010 with complete infection testing data (3 stool samples examined using the miracidium hatching test and 3 Kato-Katz slides examined) (S2 Table).

**Table 3:** The association between night soil use and *S. japonicum* infection in 36 villages in Sichuan, China, 2007 and 2010.

	**Unadjusted OR (95% CI)^a^**	**Adjustment A^b^ OR (95% CI)^a^**	**Adjustment B^c^ OR (95% CI)^a^**
**2007 (n = 2,005)**			
Village night soil use^d^			
Very low	1.00	1.00	1.00
Low	5.32 (1.95, 14.51)	7.14 (2.18, 23.38)	5.67 (1.98, 16.22)
Medium	4.69 (1.96, 11.20)	8.12 (2.83, 23.28)	8.50 (2.85, 25.35)
High	5.29 (2.04, 13.70)	10.38 (3.39, 31.82)	10.80 (3.25, 35.87)
Test for trend^e^	*p* = 0.106	*p* = 0.010	*p* = 0.009
Household night soil use			
No	1.00	1.00	1.00
Yes	0.96 (0.72, 1.29)	0.98 (0.72, 1.33)	0.92 (0.65, 1.30)
**2010 (n = 1,365)**			
Village night soil use^d^			
Very low	1.00	1.00	1.00
Low	0.98 (0.43, 2.25)	0.95 (0.44, 2.08)	1.45 (0.65, 3.24)
Medium	0.80 (0.27, 2.35)	0.78 (0.26, 2.40)	1.32 (0.41, 4.23)
High	0.30 (0.06, 1.54)	0.35 (0.06, 2.15)	0.31 (0.06, 1.58)
Test for trend^e^	*p* = 0.370	*p* = 0.388	*p* = 0.536
Household night soil use			
No	1.00	1.00	1.00
Yes	0.80 (0.46, 1.38)	0.76 (0.43, 1.33)	0.68 (0.37, 1.25)

^a^Odds ratios and 95% CIs were estimated using a multi-level fixed-effect logistic regression model. All models accounted for unmeasured within-village correlation.

^b^Adjusted for age (categorized in 10-year increments), sex and county of residence.

^c^Adjusted for all variables in Adjustment A as well as whether anyone in the household owned bovines, village bovine density (the mean number of bovines per household), household and village SES, the area cultivated by household members in the past year and village agricultural intensity (the mean area cultivated per household in the past year).

^d^Village-level night soil use describes the mean buckets of night soil applied per household in the village, calculated excluding the index household. It is categorized by quartiles: very low (0–19 buckets), low (20–33 buckets), medium (34–68 buckets) and high (69–245 buckets).

^e^Tests for trend were conducted by modeling the categorical variable as ordinal.

**Intervention attributable ratio**. The estimated intervention attributable ratio was 0.51 (95% CI 0.29, 0.88) in 2007 and 1.44 (95% CI 0.85, 2.01) in 2010. If night soil use were eliminated, we estimate infection prevalence would be reduced by 49% of the observed infection prevalence in 2007. No reductions in infection prevalence were estimated if the same intervention were to have occurred in 2010.

### Night soil from unimproved and improved sanitation sources

**Figure 1:**
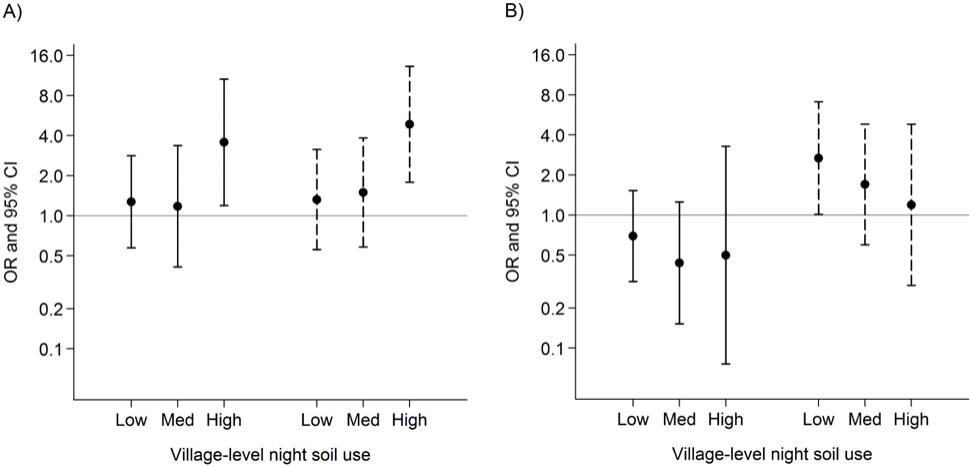
The relationship between *S. japonicum* infection and night soil from improved and unimproved sources. Figure shows odds ratios (points) and 95% confidence intervals (lines) estimating the association between *S. japonicum* infection and night soil application from improved sanitation systems (dashed lines) and unimproved sources (solid lines) in 2007 (A) and 2010 (B). A household was classified as having improved sanitation if they reported having a working anaerobic biogas digester or triple compartment septic tank. Models were adjusted for age (categorized in 10-year increments), sex, county of residence and household night soil use. Village night soil use was defined as the average quantity of night soil used by all households in the village excluding the index household and was categorized by quartiles, with the lowest quartile serving as the reference group. Unimproved night soil categories: very low (0–10 buckets), low (11–22 buckets), medium (23–48 buckets) and high (49–244 buckets). Improved night soil categories: very low (0 buckets), low (0.1–2 buckets), medium (3–11 buckets) and high (12–100 buckets).

In 2007, the amount of night soil applied in a village from both improved and unimproved sources applied was positively associated with *S. japonicum* infection (Fig. 1). The prevalence of *S. japonicum* infection was higher in areas where night soil from improved or unimproved sources were in the highest vs. lowest quartiles (unimproved sources OR 3.56, 95% CI: 1.19, 10.63; improved sources OR 4.87, 95% CI 1.79, 13.26). The positive trend was significant across quartiles of night soil from improved sources (test for trend, *p* = 0.008) but not for unimproved sources (test for trend, *p* = 0.118). In 2010, there was no association between *S. japonicum* infection and village-level night soil from improved or unimproved sources.

## Discussion

This study is, to our knowledge, the first epidemiological study to estimate the association between human schistosomiasis and the use of human waste as an agricultural fertilizer. Human waste is used as an agricultural fertilizer in many areas including Asia [31, 32], West Africa [33], Central America and Northern Europe [1]. The global extent of this practice is not well documented. We found approximately half of households in our study applied human waste to their crops. A study in Vietnam estimated over 90% of farmers in the study region used night soil [32]. In our study, night soil was applied to all major crops, in both growing seasons and by household across the socio-economic spectrum. Night soil was extracted from improved and unimproved sanitation systems. Chemical fertilizer use was ubiquitous, demonstrating night soil is used in conjunction with rather than in place of chemical fertilizers.

We found a strong positive association between the amount of night soil used in a village and human schistosomiasis in 2007, but not in 2010. We estimated a 49% reduction in schistosomiasis prevalence if night soil were eliminated using data from 2007 and the descriptive models suggested an exposure-response relationship, but we estimated no reduction in 2010. We explored several possible explanations for these contradictory findings.

First, we suspected that increased access to improved sanitation in 2010 may have reduced *S. japonicum* egg content in night soil and, subsequently, the risks of this practice. But this does not appear to be the case. While access to improved sanitation increased, the fraction of night soil that came from unimproved sources changed minimally from 2007 to 2010 (from 79% to 72%). Further, the use of night soil from unimproved sources, which we suspect to be most likely to contain *S. japonicum* eggs, was positively associated with *S. japonicum* infection in 2007 but not 2010.

Second, unmeasured confounding may either lead to a spurious association between village night soil and schistosomiasis infection in 2007 or mask a true association in 2010. We attempted to control for a large set of potential confounders including agricultural practices, SES and demographic variables. The similarity of estimates across models using a limited set of confounders (age, sex and county of residents) vs. the full set of confounders suggests the additional agricultural and socio-economic variables were not strong confounders. To explore this further, we conducted a sensitivity analysis to evaluate the impact of additionally controlling for the types of crops planted in each village. We re-ran our categorical analyses, additionally adjusting for the percent of cultivated land dedicated to rice at the village level, as well as an indicator as to whether the household planted rice in the past year. Rice was included because moist rice paddies can provide snail habitat and farmers often have contact with surface water during rice planting. There were no meaningful changes in estimates or inference with the addition of these potential confounders (S3 Table). While we cannot rule out unmeasured confounding, our findings are stable across three sets of potential confounders.

Third, the inconsistent associations between night soil use and infection may reflect shifts in the relative importance of different schistosome sources in a changing environment. There are a number of sources of schistosomes that can contribute to human infection risk including both within-village sources and parasites imported from external sources [16]. In this study, we focused on a single internal source of schistosomes: night soil use within the village. Other potential sources of schistosomes include bovines, which produce large quantities of waste and are used to plow fields, placing them in close proximity to irrigation ditches where snails live. Bovines have been shown to play an important role in *S. japonicum* transmission in the lakes region of China [34], and we previously found high prevalence of bovine infections in our study region [17]. Other non-human hosts as well as open defecation practices and leaky stool pits may also serve as internal sources of schistosomes, although we assume their contributions are modest. We know little of external sources, but they may include larval stages transported over hydrological pathways and/or eggs imported by mobile human or animal hosts. There were a number of notable changes in the study region from 2007 to 2010: a modest decline in human infection prevalence and intensity, a decline in the frequency and quantity of reported night soil use, a 39% decline in bovine ownership, an increase in SES and ongoing schistosomiasis control activities—changes that could have shifted the dominant drivers of human infection. Hence one interpretation of the lack of association between night soil use and infection in 2010 is that human schistosomiasis infection risk in 2010 is dominated by external sources and/or bovines, due to the decline in night soil use and human infections from 2007 to 2010. We suspect schistosomiasis transmission at low infection intensities is highly stochastic—when parasite loads are low, small environmental and behavior changes can lead to substantial shifts in transmission dynamics—creating both challenges and opportunities for elimination.

Forth, we assumed that all infections were acquired recently and therefore night soil use in the past year is a reasonable exposure window, but treatment failure due to non-compliance or resistance may have led to violations of these assumptions [35]. All participants testing positive for *S. japonicum* in this study were promptly treated and county health officials implemented several mass and targeted chemotherapy campaigns during the study period. While there is no conclusive evidence that praziquantel resistance is occurring in China, we have previously found the same individuals repeatedly test positive for *S. japonicum* and it is possible these individuals harbor uncured infections [36, 37]. We do not know to what extent treatment failure is occurring, or the extent to which it is caused by praziquantel resistance vs. non-compliance due to populations tiring of repeated treatment campaigns. This issue warrants further examination. If treatment failure is occurring, it is possible observed infections may be due to exposures outside of the one-year exposure window we considered.

We were surprised to find that night soil from households with improved sanitation was positively associated with *S. japonicum* infection prevalence in 2007. If night soil use does contribute to *S. japonicum* infection risk, effluent from both improved and unimproved sources may be contributing to this risk. Controlled studies have demonstrated that anaerobic biogas digesters, the predominant improved sanitation system in the region, remove viable *S. japonicum* eggs from effluent through chemical inactivation and sedimentation [18, 19]. However, ambient temperatures can impact biogas digestion, particularly in household level systems such as the ones found in our study region with low thermal mass [38] and the length of *S. japonicum* survival in biogas systems increases at lower temperatures [19]. Anecdotally, some farmers in our study villages report little or no methane production in the winter months, which suggests night soil extracted in the winter or spring may not be sufficiently digested and may contain viable *S. japonicum* eggs. Newer rural development programs have promoted construction of biogas digesters that serve multiple families. The greater size and waste input may lead to year round removal of viable *S. japonicum* eggs from effluent.

### Toward permanent reductions in schistosomiasis

While access to antihelminthic treatment remains a major global challenge [39], programs such as China’s national schistosomiasis control program and the multinational Schistosomiasis Control Initiative have greatly reduced morbidity and infection intensities in the regions where these interventions have been implemented [13, 40]. In Sichuan, for example, a survey of 20 villages conducted in 2000 found *S. japonicum* infection prevalence was 29% and mean infection intensity, 26 EGP, with village prevalence and intensity as high as 73% and 104 EPG [9]. Today, schistosomiasis transmission has been interrupted in 41 counties in Sichuan [41]. The study sites described here provide an example of the schistosomiasis transmission pockets that remain—areas where few individuals are infected and those that are infected are shedding few parasites.

We suspect the dynamics of transmission in such areas differ from areas with high infection prevalence and intensity [42]. Despite the low infection prevalence and intensities, schistosomiasis in our study sites has been remarkably robust to efforts to eliminate schistosomiasis infections. Following the reemergence of schistosomiasis in the region, the study area has been the focus of control efforts including snail control through the application of molluscicides to snail habitat (applied up to 3 times annually from 2007 to 2010), health education, improved sanitation construction and both mass and targeted chemotherapy in addition to the treatment provided following positive infection testing in our infection surveys. These control activities have yielded only modest declines in infection prevalence and intensity—infection prevalence declined from 8.4% in our study population in 2007 to 7.4% in 2010 and infection intensity from 2.1 to 1.3 EPG—a decline far less dramatic than observed following the initiation of control programs in highly endemic areas. In these areas of disease emergence and persistence, interventions that can permanently interrupt the transmission cycle are needed.

Improvements to sanitation have been one of the strategies adopted by the Chinese schistosomiasis control program, and the World Health Organization and others have recognized the importance of improvements to water and sanitation in the control of human helminthiases [10, 14, 43]. Improvements to sanitation infrastructure can reduce the potential of a schistosome to complete its life cycle by preventing human fecal waste from contaminating snail habitat and thereby preventing schistosome infections in snails. The Chinese government has subsidized sanitation improvements in schistosomiasis endemic areas and the increase in access can be observed in the study sites: improved sanitation access increased from 16.5% of households in 2007 to 26.7% in 2010. However, the extraction of human waste from stool pits to apply as an agricultural fertilizer may compromise sanitation investments.

### Conclusions

In our study of 36 villages in rural China, we found that human waste is commonly used as an agricultural fertilizer. This practice was strongly associated with schistosomiasis prevalence in 2007 but not in 2010. The inconsistent relationship may be due to the stochastic nature of schistosomiasis transmission on the margins of disease elimination. Our findings suggest a possible link between schistosomiasis and the use of human waste as an agricultural fertilizer. Further evaluation of the relationship between human helminthiases and night soil use is warranted, particularly in areas where helminthiases persists despite disease control programs.

## Supporting Information

S1 Checklist STROBE checklist. (PDF)

S1 Table Demographic characteristics of residents in 36 villages in Sichuan, China surveyed in 2007 and 2010. (PDF)

S2 Table The association between night soil use and *S. japonicum* infection in 36 villages in Sichuan, China, 2007 and 2010 among people with complete infection testing. (PDF)

S3 Table The association between night soil use and *S. japonicum* infection in 36 villages in Sichuan, China, 2007 and 2010, adjusted for rice cultivation. (PDF)
