# Supplementary material for: Associations between Schistosomiasis and the Use of Human Waste as an Agricultural Fertilizer in China
Source: PLoS Negl Trop Dis. 2015 Jan 15;9(1):e0003444. doi: 10.1371/journal.pntd.0003444 (PMC4295866; doi:10.1371/journal.pntd.0003444)
Supplement: S1 Table — (PDF) [file pntd.0003444.s002.pdf]

Table S1. Demographic characteristics of residents in 36 villages in Sichuan, China surveyed in 2007 and 2010.

	2007		2010	
	Census	Tested for infection	Census	Tested for infection
	No.	No. (%)	No.	No. (%)
Age				
< 15 years	339	186 (55)	143	78 (55)
15 - 24 years	263	99 (38)	179	42 (23)
25 - 34 years	394	249 (63)	211	88 (42)
35 - 44 years	763	536 (70)	584	355 (61)
45 - 54 years	590	473 (80)	477	333 (70)
≥ 55 years	542	462 (85)	693	469 (68)
Sex				
Female	1431	1023 (71)	1166	727 (62)
Male	1460	982 (67)	1121	638 (57)
County of residence				
County 1	1704	1141 (67)	1409	762 (54)
County 2	1187	864 (73)	878	603 (69)
