# Supplementary material for: Associations between Schistosomiasis and the Use of Human Waste as an Agricultural Fertilizer in China
Source: PLoS Negl Trop Dis. 2015 Jan 15;9(1):e0003444. doi: 10.1371/journal.pntd.0003444 (PMC4295866; doi:10.1371/journal.pntd.0003444)
Supplement: S2 Table — (PDF) [file pntd.0003444.s003.pdf]

Table S2. The association between night soil use and *S. japonicum* infection in 36 villages in Sichuan, China, 2007 and 2010 among people with complete infection testing.

	Undadjusted OR (95% CI)[a]		Adjustment A[b] OR (95% CI)[a]		Adjustment B[c] OR (95% CI)[a]	
**2007 (n=1,577)**						
Village night soil use[d]						
Very low	1.00		1.00		1.00	
Low	5.73	(1.19 - 27.69)	9.36	(1.77 - 49.45)	6.83	(1.33 - 35.01)
Medium	4.88	(1.07 - 22.37)	10.42	(2.06 - 52.66)	9.04	(1.71 - 47.89)
High	5.64	(1.13 - 28.13)	13.85	(2.52 - 76.25)	11.28	(1.95 - 65.11)
Test for trend[e]	*p*=	0.188	*p*=	0.018	*p*=	0.028
Household night soil use						
No	1.00		1.00		1.00	
Yes	0.88	(0.68 - 1.13)	0.89	(0.68 - 1.16)	0.86	(0.62 - 1.18)
**2010 (n=747)**						
Village night soil use[d]						
Very low	1.00		1.00		1.00	
Low	1.00	(0.38 - 2.63)	0.94	(0.35 - 2.51)	1.31	(0.43 - 4.03)
Medium	1.19	(0.38 - 3.70)	1.29	(0.45 - 3.68)	2.36	(0.69 - 8.07)
High	0.18	(0.03 - 1.17)	0.27	(0.03 - 2.03)	0.27	(0.04 - 1.73)
Test for trend[e]	*p*=	0.579	*p*=	0.785	*p*=	0.977
Household night soil use						
No	1.00		1.00		1.00	
Yes	0.81	(0.43 - 1.51)	0.77	(0.40 - 1.47)	0.72	(0.35 - 1.49)

[a]Odds ratios and 95% CIs were estimated using a multi-level fixed-effect logistic regression model. All models accounted for unmeasured within-village correlation.
[b]Adjusted for age (categorized in 10-year increments), sex and county of residence.
[c]Adjusted for all variables in Adjustment A as well as whether anyone in the household owned bovines, village bovine density (the mean number of bovines per household), household and village SES, the area cultivated by household members in the past year and village agricultural intensity (the mean area cultivated per household in the past year).
[d]Village-level night soil use describes the mean buckets of night soil applied per household in the village, calculated excluding the index household. It is categorized by quartiles: very low (0-19 buckets), low (20-33 buckets), medium (34-68 buckets) and high (69-245 buckets).
[e]Tests for trend were conducted by modeling the categorical variable as ordinal.

Analysis includes the 1,577 individuals in 2007 and 747 individuals in 2010 with complete infection testing results. These individuals submitted three stool samples, each was examined using the miracidium hatching test, and one stool sample was examined using three Kato-Katz slides.
