# Supplementary material for: Associations between Schistosomiasis and the Use of Human Waste as an Agricultural Fertilizer in China
Source: PLoS Negl Trop Dis. 2015 Jan 15;9(1):e0003444. doi: 10.1371/journal.pntd.0003444 (PMC4295866; doi:10.1371/journal.pntd.0003444)
Supplement: S3 Table — (PDF) [file pntd.0003444.s004.pdf]

Table S3. The association between night soil use and *S. japonicum* infection in 36 villages in Sichuan, China, 2007 and 2010, adjusted for rice cultivation.

	Adjustment C[a] OR (95% CI)[b]	
**2007 (n=2,005)**		
Village night soil use[c]		
Very low	1.00	
Low	5.38	(1.99 - 14.54)
Medium	8.72	(2.92 - 26.03)
High	11.63	(3.24 - 41.73)
Test for trend[d]	*p*=	0.008
Household night soil use		
No	1.00	
Yes	0.93	(0.66 - 1.32)
**2010 (n=1,365)**		
Village night soil use[c]		
Very low	1.00	
Low	1.38	(0.67 - 2.86)
Medium	1.41	(0.45 - 4.42)
High	0.33	(0.07 - 1.64)
Test for trend[d]	*p*=	0.572
Household night soil use		
No	1.00	
Yes	0.68	(0.37 - 1.23)

[a]Odds ratios and 95% CIs were estimated using a multi-level fixed-effect logistic regression model. All models accounted for unmeasured within-village correlation.
[b]Adjusted for all variables in Adjustment A (age, sex, county of residence), Adjustment B (whether anyone in the household owned bovines, village bovine density, household and village SES, the area cultivated by household members in the past year and village agricultural intensity), as well as whether anyone in the household planted rice in the past year, and the percent of total cultivated area in the village devoted to rice cultivation.
[c]Village-level night soil use describes the mean buckets of night soil applied per household in the village, calculated excluding the index household. It is categorized by quartiles: very low (0-19 buckets), low (20-33 buckets), medium (34-68 buckets) and high (69-245 buckets).
[d]Tests for trend were conducted by modeling the categorical variable as ordinal.
